# Ureteric Injury From an Indwelling Catheter in the Context of an Undiagnosed Duplex Ureter in a Labouring Woman: A Case Report

**DOI:** 10.7759/cureus.104410

**Published:** 2026-02-27

**Authors:** Kira Sklar

**Affiliations:** 1 Obstetrics and Gynaecology, Sutherland Hospital, Sydney, AUS

**Keywords:** caesarean section, duplex collecting system, duplicated ureter, obstetrics, ureteric injury, ureteric rupture, ureteric stent

## Abstract

Indwelling urinary catheter (IDC) insertion is routine in obstetric practice and is considered low risk. Inadvertent ureteric catheterisation is rare and has been reported only in a few patients with complex backgrounds, mainly neurogenic bladder. This report describes a case of iatrogenic ureteric injury secondary to IDC insertion in an otherwise well woman, who was later found to have an undiagnosed duplex renal collecting system. Interestingly, an antenatal ultrasound in this case detected a fetal duplex renal collecting system.

The case describes a woman in her thirties in spontaneous labour who had an IDC inserted due to concurrent epidural analgesia. She was noted to have minimal urine output after IDC insertion. She required an emergency caesarean section for a prolonged second stage and fetal tachycardia, and the bladder was noted to be oedematous and high during entry. Further imaging with computerized tomography following the caesarean section demonstrated the duplex collecting system with evidence of ureteric rupture of the lower pole moiety. The IDC was seen to be malpositioned, passing into the left ureter. The patient then required emergency cystoscopy with stent insertion. This case highlights a rare but serious complication related to routine IDC insertion. Consideration of maternal imaging when fetal renal anomalies are detected may be useful in diagnosing maternal duplex systems; however, current evidence does not support this practice.

## Introduction

Insertion of indwelling urinary catheters (IDC) is a commonly performed bedside procedure. It is considered a low-risk procedure and most often proceeds without issue [1]. In labour, there are multiple scenarios when IDC insertion is routinely offered, including postpartum haemorrhage management and with epidural insertion [2]. In very rare cases, IDCs have inadvertently been inserted into the ureter, leading to complications such as hydronephrosis or ureteric rupture [1]. However, in the majority of the previously reviewed cases, patients had risk factors such as long-term IDC use for neurogenic bladder. This review describes a case of a young, otherwise well woman, who had an undiagnosed left duplex collection system.

A duplex system refers to a common renal tract abnormality wherein one kidney has two separate, noncommunicating renal pelvises. Duplication can be incomplete, wherein two pelvises are present but share a single ureter that inserts into the bladder, or complete wherein each pelvis has its own ureter that each inserts separately into the bladder [3]. This patient was found to have a complete duplication, with one moiety in close proximity to the internal urethral orifice. It was suspected that this anatomical variation led to inadvertent catheterisation of her duplex left ureter while in labour, which resulted in ureteric injury.

## Case presentation

This patient is a primiparous woman in her thirties who presented to the delivery suite in spontaneous labour at 39+1 weeks gestation. This was a planned spontaneous pregnancy. Her antenatal issues included diet-controlled gestational diabetes and antenatal ultrasounds demonstrating a left fetal duplex kidney. She had no previous medical or surgical history and no relevant family history.

The patient presented to the birth unit in spontaneous labour. At initial review, she was two centimetres dilated on examination. Approximately two hours later, the woman requested an epidural block (EDB) for analgesia. As is done routinely with EDB insertion, she had an IDC inserted at the same time, which was noted to drain clear urine. The woman reached full dilation of the cervix approximately 12 hours after her initial exam. After an hour of passive descent followed by an hour of pushing, the presenting part of the fetus remained at the level of the ischial spines with minimal descent while pushing. Cardiotocography (CTG) also demonstrated an increasing fetal heart rate after having previously been reassuring.

The patient proceeded to have an emergency caesarean section for prolonged second stage and fetal tachycardia. At entry into the abdomen, the patient’s bladder was noted to be oedematous and high, and the IDC balloon was not palpable in the bladder. The procedure was paused, and a new IDC was inserted without resistance. There was no haematuria. The procedure was then resumed, and the bladder was well reflected.

**Figure 1:**
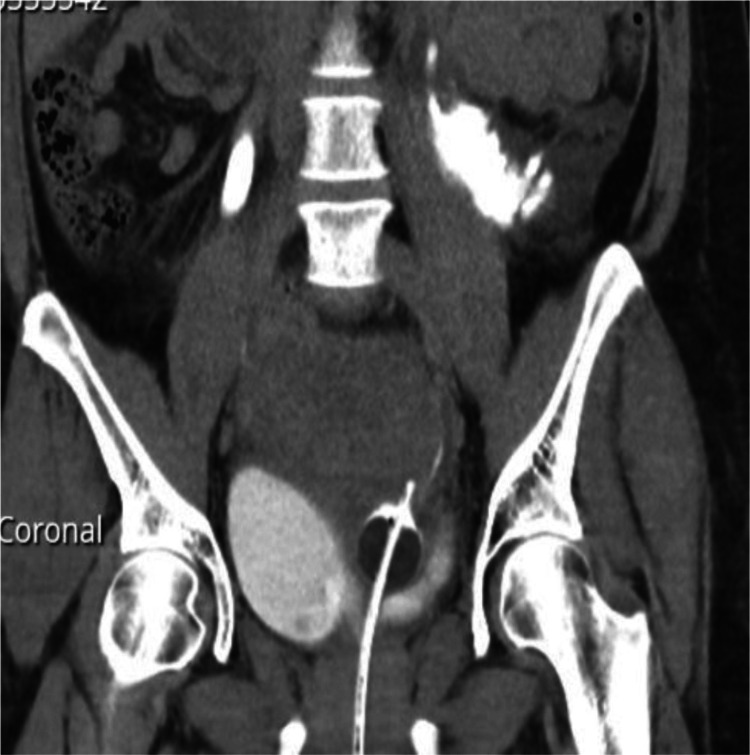
Coronal CT intravenous pyelogram demonstrating urinary catheter malposition, with catheter appearing to pass into the left distal ureter.

**Figure 2:**
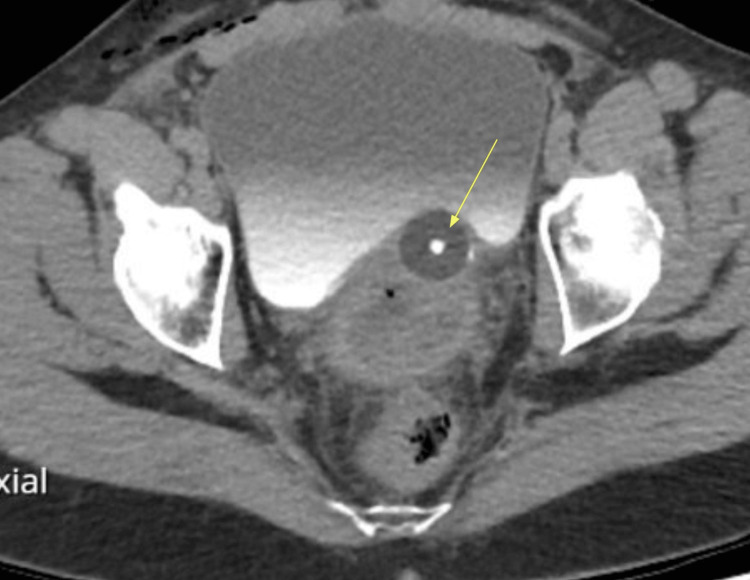
Axial CT intravenous pyelogram demonstrating urinary catheter malposition outside of the urinary bladder.

At the end of the procedure, it was noted that there was minimal IDC output, and the IDC was replaced once again after confirmation that the tubing was not kinked and that the patient was appropriately hydrated. After a further period of minimal urine output with appropriate hydration, additional investigations were organised. The patient had a computerized tomography intravenous pyelogram scan (CT IVP), which demonstrated a distended bladder and showed that the urinary catheter was malpositioned with the balloon abutting the posterior margin of the left side of the bladder, with the catheter appearing to pass into the distal left ureter. A duplex left-collected system was noted, wherein two left ureteric orifices each opened into the bladder. The upper pole was intact. The lower pole was noted to be dilated and poorly defined with extravasation of contrast, which was suggestive of rupture (Figures 1, 2).

**Figure 3:**
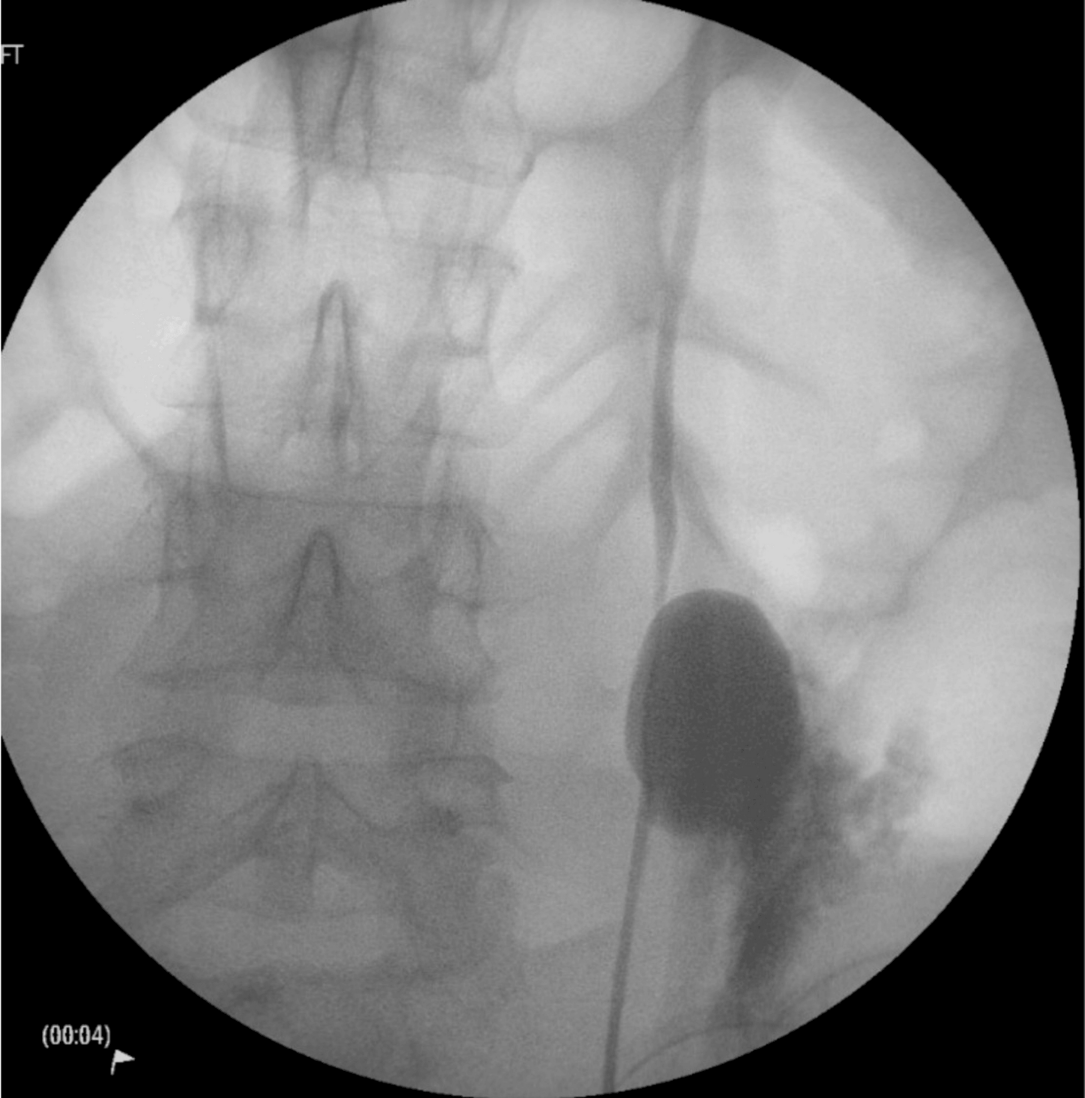
Intraoperative pyelogram demonstrating contrast extravasation secondary to ureteric injury.

**Figure 4:**
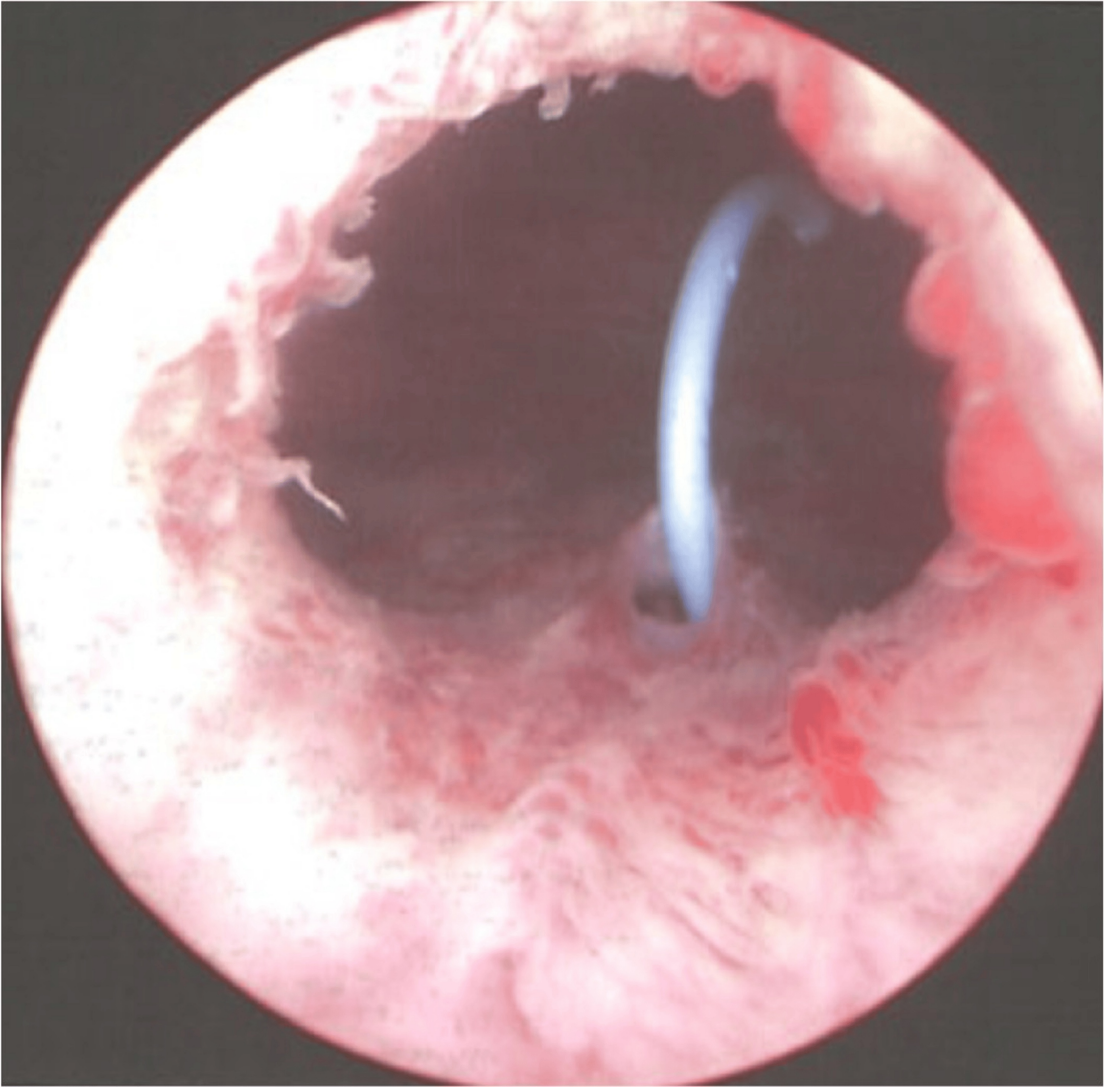
Cystoscopy image demonstrating rupture of the left ureter. The lower moiety ureteric orifice was in close proximity to urethra, which can be seen here. The proximity contributed to the inadvertent ureteric catheterisation.

The Urology team was consulted after this finding. After discussion with the patient involving open disclosure and informed consent, the Urology team then performed an emergency cystoscopy, left retrograde pyelogram, and insertion of a left JJ stent. At the start of the procedure, the patient’s IDC was removed, and she was noted to have 850mL retained urine. During cystoscopy, the lower moiety ureteric orifice was seen to be unusually low and in close proximity to the internal urethral orifice. The impression was that, due to this anatomical variation, the IDC had most likely been inserted into the lower pole of the ureteric orifice, which led to disruption of the distal ureter, while the upper pole remained intact (Figures 3, 4). A 4.8 French JJ stent was inserted into the lower pole moiety of the left collecting system.

The patient had a new IDC inserted following stenting, which was draining urine well. She was discharged at four days postpartum with a plan to keep the IDC in situ for two weeks, and to have a cystogram and urology follow-up for a trial of void. On discharge, it was discussed that she would likely have the stent out in 2 months time after a renogram, if her urine was shown to be draining well. She was reassured that this injury was unlikely to cause long-term issues.

## Discussion

Duplex collecting systems are a relatively common renal anomaly, and many affected individuals are asymptomatic lifelong and therefore do not receive diagnoses [4]. Duplex systems are more common among women (65% of cases) and appear to have a genetic component [4].

Ureteral rupture is a result of pressure in the collecting system exceeding a critical level [5]. Most commonly, rupture can occur as a result of nephrolithiasis or ureteric structures. The gravid uterus adds to the pressure of the ureter, but only in very rare instances has this been shown to lead to ureteric rupture on its own [5]. The renal system undergoes hormonal and anatomical changes during pregnancy, including hydronephrosis and hydroureter, which can also contribute to the risk of ureteric rupture [6].

In this case, antenatal imaging did not demonstrate maternal hydronephrosis or hydroureter, and the rupture was suspected to be a result of iatrogenic trauma from IDC insertion. This was related to an undiagnosed duplex collecting system, with each left ureter opening separately into the bladder. Based on imaging and cystoscopy, the lower pole was in close proximity to the internal urethral opening, which made the inadvertent insertion into the ureter more likely. Interestingly, for this patient, a fetal duplex system was detected antenatally. There is a heritable component to a duplex renal collecting system; however, the mother did not have any ultrasound imaging of her own renal tracts prior to suspicion of renal tract injury. It is worth considering whether fetal renal anomalies detected on ultrasound should prompt maternal renal sonography; however, current evidence does not recommend this as duplex systems are most often asymptomatic and of little consequence [7].

## Conclusions

This case demonstrates a rare but significant complication of routine indwelling urinary catheter insertion. While IDC insertion is considered a low-risk bedside procedure, anatomical variants may increase susceptibility to malposition or urinary tract injury. In obstetric patients, the gravid uterus as well as physiological hydronephrosis and hydroureter may also contribute to this risk. It is worth considering whether identification of fetal renal tract anomalies should prompt maternal renal tract imaging; however, current evidence does not recommend this practice.

Persistent low urinary output following catheterisation or unexpected intraoperative findings such as an oedematous bladder without a palpable balloon should prompt early consideration of imaging once common causes of low output, such as dehydration, hypotension, or kinking of the catheter tubing, have been excluded. In this case, early urological involvement was essential in minimising long-term sequelae for the patient.
